# The genetics of dystonia: new twists in an old tale

**DOI:** 10.1093/brain/awt138

**Published:** 2013-06-17

**Authors:** Gavin Charlesworth, Kailash P. Bhatia, Nicholas W. Wood

**Affiliations:** 1 Department of Molecular Neuroscience, UCL Institute of Neurology, Queen Square, London, WC1N 3BG, UK; 2 Sobell Department of Motor Neuroscience and Movement Disorders, UCL Institute of Neurology, Queen Square, London, WC1N 3BG, UK

**Keywords:** dystonia, genetics, molecular mechanisms, clinical phenotype

## Abstract

Dystonia is a common movement disorder seen by neurologists in clinic. Genetic forms of the disease are important to recognize clinically and also provide valuable information about possible pathogenic mechanisms within the wider disorder. In the past few years, with the advent of new sequencing technologies, there has been a step change in the pace of discovery in the field of dystonia genetics. In just over a year, four new genes have been shown to cause primary dystonia (*CIZ1*, *ANO3*, *TUBB4A* and *GNAL*), *PRRT2* has been identified as the cause of paroxysmal kinesigenic dystonia and other genes, such as *SLC30A10* and *ATP1A3*, have been linked to more complicated forms of dystonia or new phenotypes. In this review, we provide an overview of the current state of knowledge regarding genetic forms of dystonia—related to both new and well-known genes alike—and incorporating genetic, clinical and molecular information. We discuss the mechanistic insights provided by the study of the genetic causes of dystonia and provide a helpful clinical algorithm to aid clinicians in correctly predicting the genetic basis of various forms of dystonia.

## Introduction

New ‘next generation’ sequencing technologies have massively increased the speed of genetic discoveries in recent years and this has made itself felt in the field of dystonia research as in many others. In just over a year, four new genes have been shown to cause primary dystonia (*CIZ1*, *ANO3*, *TUBB4A* and *GNAL*) (Charlesworth *et al.*, 2012; Fuchs *et al.*, 2012; Hersheson *et al.*, 2012; Lohmann *et al.*, 2012; Xiao *et al.*, 2012); *PRRT2* has been identified as the cause of paroxysmal kinesigenic dystonia, and other genes (Wang *et al.*, 2011), such as *SLC30A10* and *ATP1A3*, have been linked to more complicated forms of dystonia or new phenotypes (Heinzen *et al.*, 2012; Quadri *et al.*, 2012; Rosewich *et al.*, 2012; Tuschl *et al.*, 2012). The identification of some of these genes strengthens the evidence for long-suspected molecular culprits in the pathophysiology of dystonia (e.g. dopaminergic transmission and transcription abnormalities), whereas others have highlighted less commonly implicated mechanisms that can lead to the disease (e.g. ion channel, microtubular or synaptic dysfunction). In this review, we provide a comprehensive overview of the major forms of dystonia for which the genetic cause is now known, with a particular emphasis on the primary, paroxysmal and major ‘dystonia-plus’ syndromes. Genetic, clinical and, where possible, molecular information are given and a helpful clinical algorithm is provided to aid clinicians in correctly predicting the genetic basis of various forms of dystonia.

The dystonias are a heterogenous group of hyperkinetic movement disorders, characterized by involuntary sustained muscle contractions affecting one or more sites of the body, which lead to twisting and repetitive movements or abnormal postures of the affected body part. It is the third most common movement disorder worldwide (Fanh *et al.*, 1988; Geyer and Bressman, 2006; Defazio *et al.*, 2007; Breakefield *et al.*, 2008). Approximately 70 000 people are affected by dystonia in the UK alone, including some 8000 children and adolescents (Paudel *et al.*, 2012). Affected individuals can suffer considerable physical and psychosocial distress, which has been demonstrated to have a significant impact on their quality of life (Skogseid *et al.*, 2007; Soeder *et al.*, 2009; Zoons *et al.*, 2012). The pathophysiology of the disorder is poorly understood at present. In all probability, it is heterogeneous and arises from a dysfunction of the various central neural circuits that control and coordinate voluntary movements, such as those found in the basal ganglia, the cerebellum, the sensorimotor cortex, and the interactions between these three regions of the brain (Fahn, 1988; Quartarone *et al.*, 2008; Argyelan *et al.*, 2009; Teo *et al.*, 2009).

## Classification of dystonia

The classification of the dystonia is complex and not entirely satisfactory. Several approaches or systems operate in parallel. Clinically, the dystonias are usually classified according to one of four major variables: (i) age of onset (early onset versus adult onset); (ii) distribution of affected body parts (focal, multifocal, segmental or generalized); (iii) the underlying cause (primary, secondary or heredodegenerative); or (iv) special clinical features (paroxysmal, exercise-induced, task-specific or DOPA-responsive) (Albanese *et al.*, 2011). The current European Federation of Neurological Societies recommended classification scheme is based on this approach (Table 1) (Albanese *et al.*, 2011).

**Table 1:** Classification of dystonia, based on the European Federation of Neurological Societies current scheme

**By aetiology**	
1. Primary dystonia	
1.1 Primary pure dystonia	Torsion dystonia is the only clinical sign (apart from tremor) and there is no identifiable exogenous cause or other inherited or degenerative disease
1.2 Primary plus dystonia	Torsion dystonia is a prominent sign but is associated with another movement disorder, for example myoclonus or parkinsonism. There is no evidence of neurodegeneration.
1.3 Primary paroxysmal dystonia	Torsion dystonia occurs in brief episodes with normalcy in between. Three main forms are known depending on the triggering factor.
2. Heredodegenerative dystonia	Dystonia is a feature, among other neurological signs, of a heredodegenerative disorder, such as Wilson's disease
3. Secondary dystonia	Dystonia is a symptom of an identified neurological condition, such as a focal brain lesion, exposure to drugs or chemicals, e.g. dystonia because of a brain tumour, off-period dystonia in Parkinson's disease.
**By age at onset**	
1. Early onset (<30 years of age)	Usually starts in a leg or arm and frequently progresses to involve other limbs and the trunk
2. Late onset	Usually starts in the neck (including the larynx), the cranial muscles or one arm. Tends to remain localized with restricted progression to adjacent muscles
**By distribution of affected body parts**	
1. Focal	Single body region (e.g. writer’s cramp, blepharospasm)
2. Segmental	Contiguous body regions (e.g. cranial and cervical, cervical and upper limb)
3. Multifocal	Non-contiguous body regions (e.g. upper and lower limb, cranial and upper limb)
4. Generalized	Both legs and at least one other body region (usually one or both arms)
5. Hemidystonia	Half of the body (usually secondary to a lesion in the contralateral basal ganglia)

From a genetic point of view, hereditary dystonia can be classified either by the gene causing the condition, where it is known, or by reference to one of the ever expanding list of dystonia loci, of which there are currently 23 (Table 2). The system of DYT loci is particularly unsatisfactory. The system was designed to indicate genomic regions that had been linked to a specific hereditary disorder, but where the actual causative gene was not yet known (Kramer *et al.*, 1990). DYT loci were assigned in chronological order based on the appearance of reports in the medical literature. In theory, once the underlying genetic cause was known, the locus was supposed to be withdrawn and the disorder merged into the entry for the cloned gene. However, in practice, this has not happened and both clinicians and researchers alike tend to use dystonia loci and genes names interchangeably, e.g. DYT1/*TOR1A* or DYT6/*THAP1*. With time, several other problems have arisen. The designation of some loci has never been replicated and is of questionable significance (e.g. DYT7 or DYT13), whereas others are known to be the result of incorrect assignations due to erroneous linkage (e.g. DYT9, DYT14 and DYT19) (Wider *et al.*, 2008; Weber *et al.*, 2011). Some DYT loci do not even designate any chromosomal location, but are based solely on the observation of a few families with a similar phenotype or mode of inheritance (e.g. DYT2) (Khan *et al.*, 2003; Zlotogora, 2004). More importantly, not all pathogenic mutations causing dystonia have been assigned to a DYT locus (e.g. mutations in *SPR*, *CIZ1* or *GNAL*), whereas some syndromes with prominent dystonic components have been assigned to loci belonging to other movement disorders (e.g. PARK13 and PARK14) or vice versa (DYT3 and DYT12).

**Table 2:** The current DYT loci with brief description of associated phenotype, gene of linkage interval (where known), mode of inheritance and OMIM reference numbers

Locus Symbol	Phenotype	Gene or linkage (if known)	Mode of inheritence	OMIM
DYT1	Early-onset primary torsion dystonia	*TOR1A*	AD	605204
DYT2	Early-onset primary dystonia with prominent cranio-cervical involvement	Not known	AR	224500
DYT3	Adult onset dystonia-parkinsonism, prevalent in the Philippines.	*TAF1*	X-linked	31420
DYT4	Whispering dystonia (adult onset spasmodic dysphonia) with generalization and ‘hobby horse’ gait	*TUBB4A*	AD	128101
DYT5a	Progressive DOPA-responsive dystonia with diurnal variation	*GCH1*	AD	128230
DYT5b	Akinetic rigid syndrome with DOPA-responsive dystonia or complex encephalopathy	*TH*	AR	191290
DYT6	Adult-onset torsion dystonia with prominent cranio-cervical and laryngeal involvement	*THAP1*	AD	602629
DYT7	Adult-onset primary cervical dystonia	18 p	AD	602124
DYT8	Paroxysmal non-kinesigenic dyskinesia	*MR-1*	AD	118800
DYT10	Paroxysmal kinesigenic dyskinesia	*PRRT2*	AD	128200
DYT11	Myoclonic dystonia (often with alcohol responsiveness)	*SGCE*	AD	159900
DYT12	Rapid onset dystonia parkinsonism and alternating hemiplegia of childhood	*ATP1A3*	AD (often *de novo*)	128235
DYT13	Early onset torsion dystonia in one Italian family	1p36.32-p36.13	AD	607671
DYT15	Myoclonic dystonia with alcohol responsiveness in one Canadian kindred	18p11	AD	607488
DYT16	Early-onset dystonia-parkinsonism	*PRKRA*	AR	612067
DYT17	Primary focal dystonia with progression in one Lebanese family	20p11.2-q13.12	AR	612406
DYT18	Paroxysmal exercise-induced dyskinesia ± epilepsy	*SLC2A1*	AD	612126
DYT20	Paroxysmal non-kinesiogenic dyskinesia 2, in one large Canadian family	2q31	AD	611147
DYT21	Adult-onset mixed dystonia with generalization in one Swedish family	2q14.3-q21.3	AD	614588
DYT22	Reserved, but not published	?	?	?
DYT23	Autosomal dominant, often tremulous cranio-cervical dystonia ±upper limb tremor	*ANO3*	AD	610110

DYT9, DYT14, DYT19 are not included in the table as they are now known to be synonymous with DYT18, DYT5a, and DYT19 respectively. AD = autosomal dominant; AR = autosomal recessive.

## Investigation of dystonia

The diagnosis of dystonia is, fundamentally, clinical. It relies on the presence of repetitive or sustained abnormal postures (with or without tremor) and the recognition of specific features, such as a *geste antagoniste* or overflow and mirror movements. *Geste antagoniste* refers to a voluntary manoeuvre (such as touching the face or an affected body part) that temporarily reduces the severity of dystonic posture or movements. An overflow movement is an unintentional muscle contraction that accompanies, but is anatomically distinct, from the primary dystonic movement, i.e. posturing of a hand normally unaffected by dystonia when performing tasks with the affected hand. Conversely, mirror movements are dystonic postures of a body part normally affected by dystonia when performing a motor task with a body part that is not affected by dystonia.

In general, for primary dystonia, few, if any, tests are required. The main exception to this rule is early-onset (<30 years of age) dystonia of unknown aetiology, which should always prompt consideration of a diagnosis of DOPA-responisve dystonia or Wilson’s disease, as accurate identification of these diseases at an early stage will permit the introduction of potentially life-changing treatments. Therefore, many would advocate, at the very least, a metabolic analysis that includes measurement of serum copper and caeruloplasmin and, possibly, a trial of l-DOPA in this group. In practice, an MRI and genetic testing for *TOR1A* and *THAP1* mutations are often also performed. Finally, given the recent identification of a new form of treatable dystonia caused by brain manganese deposition secondary to mutations in *SLC30A10* (Quadri *et al.*, 2012; Tuschl *et al.*, 2012), serum manganese measurement should at least be considered.

The features listed in Table 3 are those that might raise suspicion that the dystonia is not primary and trigger further investigation. The purpose of such investigations is to identify a secondary cause for the dystonia or to further elucidate the cause of dystonia presenting as part of a heredodegenerative condition. In practice, a combination of blood tests, structural imaging and selected secondary investigations are usually required to secure the diagnosis and Table 4 gives an indication of some of the investigations that may be appropriate given the aetiology under consideration. As regards neuroimaging, MRI is generally the modality of choice, although secondary CT may be required to accurately distinguish calcium from iron deposition in the basal ganglia. A dopamine transporter (DaT) scan may be useful to distinguish DOPA-responsive dystonia or rapid-onset dystonia-parkinsonism (where it will be normal) from other causes of parkinsonism with secondary dystonia (Romero-Lopez *et al.*, 2008; Zanotti-Fregonara *et al.*, 2008).

**Table 3:** Features suggestive of non-primary dystonia

Abnormal birth or perinatal history	
Dysmorphia	
Delayed developmental milestones	
Seizures	
Hemidystonia	
Sudden onset or rapidly progressive dystonia	
Prominent oro-bulbar dystonia	
The presence of another movement disorder (except tremor)	
Neurological signs suggesting involvement of other neurological systems (pyramidal signs, cerebellar signs, neuropathy, cognitive decline)	
Signs suggesting disease outside of the nervous system (hepatomegaly, splenomegaly)	

Some features that should raise suspicion that dystonia is secondary or heredodegenerative and trigger further investigation. It should be noted that some of these features are found in some types of primary dystonia, but their presence should nonetheless trigger careful consideration of a secondary dystonia or heredodegenerative disorder.

**Table 4:** Investigations used in the diagnosis of secondary and heredodegenerative dystonia with example indication

Investigation	Example indications
**Blood**	
Acanthocytes	Neuroacanthocytosis, neuronal brain iron accumulation
Alpha fetoprotein	Ataxia telangiectasia
Creatinine kinase	Neuroacanthocytosis
Copper and caeruloplasmin	Wilson’s disease, neuronal brain iron accumulation
Lactate and pyruvate	Mitochondrial disorders
Serum manganese	Dystonia with brain manganese deposition due to *SLC30A10* mutation
Serum ferritin	Neuroferritinopathy
White cell enzymes	Lysosomal storage disorders
**Urine**	
Urinary amino acids	Aminoacidaemias
24 h urinary copper	Wilson’s disease
**Neuroimaging**	
MRI	Most secondary causes, looking for structural lesions, iron/calcium deposition, caudate atropy, white matter abnormalities, etc.
Dopamine transporter (DaT) scan	Parkinsonism
**Other**	
Nerve condition tests	Spinocerebellar ataxia, neuroacanthocytosis, metachromatic leukodystrophy
Trial of l-DOPA	Early onset dystonia (<30 years of age) of unknown aetiology
Autonomic function tests	Multiple system atrophy
Slit-lamp examination	Wilson’s disease
Liver biopsy	Wilson’s disease
Muscle biopsy	Mitochondrial disorders
Electro-retinography	Neuronal brain iron accumulation
Genetic tests	See Table 7 for the genetic causes of heredodegenerative dystonias

This list is not exhaustive.

## Genetic burden in dystonia and genetic testing

Current evidence suggests that there is a significant genetic contribution to many forms of dystonia. Monogenic inheritance is most often seen in early-onset cases, where a family history can often be elicited. However, the reduced penetrance of some monogenic forms of dystonia, such as those due to mutations in *TOR1A* and *THAP1*, means that many apparently sporadic cases may also fall into this category. Furthermore, it is likely that a number of genes responsible for familial dystonia remain to be discovered, such that negative genetic testing for all currently known dystonia genes does not imply that the disorder is not genetic.

Late-onset dystonia, which represents by far the greatest number of cases, also appears to have a strong genetic basis. Studies based on the clinical examination of first-degree relatives of patients with focal dystonia have reported a risk of developing the same or another form of dystonia in the range of 23 to 36% (Waddy *et al.*, 1991; Schmidt *et al.*, 2009). Epidemiological studies have suggested that, although often apparently sporadic, adult onset dystonia may sometimes be inherited in an autosomal dominant manner, but with a marked penetrance of 12 to 15% (Waddy *et al.*, 1991; Stojanovic *et al.*, 1995; Leube *et al.*, 1997*b*). Unfortunately, this presents significant challenges for gene discovery and, at present, the genetic architecture of late onset dystonia remains largely unknown. It is possible that the use of endophenotypes, based on imaging or neurophysiological testing, to identify non-penetrant mutation carriers will help by permitting accurate linkage and segregation analyses in some of the larger kindreds (Defazio *et al.*, 2007; Kimmich *et al.*, 2011). However, multigenic inheritance, resulting from the combination of two or more genetic changes, each imparting a low to moderately increased risk of developing dystonia and acting in combination with environmental factors, may also underlie a significant proportion of the apparent heritability of late-onset dystonia. Large-scale genome-wide association studies will be helpful in dissecting out the genetic contribution in these cases.

**Figure 1:**
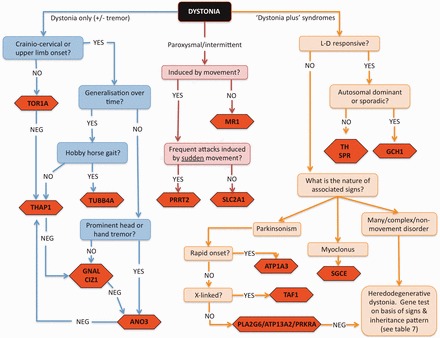
A workable strategy for identifying the likely genetic basis for some of the major forms of dystonia. Mutational screening for some genes is currently only available on a research basis.

At the present time, genetic testing is most profitably employed in familial or early-onset cases, where it is likely to have the highest yield. Despite the fact that the establishment of a molecular diagnosis rarely alters management radically, it can be helpful for patients to understand the cause of their dystonia and also bring a halt to unnecessary continued investigation, as well as allowing the physician to impart accurate information regarding the risk of recurrence in subsequent generations. A scheme for deciding which genes may be appropriate to test in which patients is provided in Fig. 1.

## Monogenic forms of dystonia

In the sections below, we present an overview of the major forms of dystonia exhibiting Mendelian inheritance for which an underlying genetic cause has been identified. Table 5 offers an overview of some of the key features of each of the various subtypes of primary dystonia where the causative gene is known.

**Table 5:** Summary of the key clinical features of some of the major forms of primary dystonia by causative gene

	Gene	Typical age range for onset	Other notable non-dystonic signs	Typical distribution of dystonia (at onset)	Generalization or progression?	Clinical clues or other special features
**Primary pure**	*TOR1A* (DYT1)	Childhood	–	Legs ≫ arms	Often Generalizes	Jewish ancestry; dystonia progressing to fixed deformity; laryngeal or cranial sparing
	*THAP1* (DYT6)	Adolescence to early adulthood	–	Laryngeal, cervical or brachial	Generalization common	Laryngeal or brachial onset with progression.
	*CIZ1*	Adult	–	Cervical	No generalization	Only reported in pure focal cervical dystonia
	*ANO3* (DYT23)	Adolescence to early adulthood	–	Craniocervical or brachial	No generalization	Prominent head, voice or arm tremor.
	*TUBB4A *(DYT4)	Adolescence to early adulthood	–	Laryngeal, craniocervical	Generalization observed	Ataxic, hobbyhorse gait; extrusional tongue dystonia; single family
	*GNAL*	Adolescence to mid-life	–	Craniocervical	Generalization observed	–
**Paraoxysmal**	*MR-1* (DYT8)	Childhood to adolescence	Choreatic dyskinesias; ballism	Limbs and face	No	Long (minutes to hours) attacks, triggered by alcohol, caffeine and emotional stress.
	*SLC2A1* (DYT18)	Childhood to adolescence	Epilepsy (see clinical clues also)	Legs	Progression to static dystonia can occur	Exercise or hunger induced. May be associated with other complex neurology: ataxia, spasticity etc, or haemolytic anaemia.
	*PRRT2* (DYT10)	Childhood to adolescence	Choreatic dyskinesias; childhood epilepsy.	Limbs (generally the limb affected is the limb that is moving)	No	Frequent, brief (seconds to minutes) attacks triggered by movement. Family history of childhood epilepsy or hemiplegic migraine
**Dystonia-plus**	*GCH1* (DYT5a)	Childhood	Parkinsonism in some	Legs	Progressive, but often becomes static later.	Diurnal variation; dramatic response to l-DOPA; may mimic a spastic paraparesis.
	*TH* (DYT5b)	Infancy to childhood	Progressive hypokinetic rigidity; complex encephalopathy in some; ‘lethargy-irritability’ crises.	Generalized but often with axial hypotonia.	Progressive without treatment	l-DOPA response related to phenotype: response tends to be less good in those with complex encephalopathy.
	*SPR*	Infancy to childhood	Many: motor/speech delay, axial hypotonia, parkinsonism, dysarthria, autonomic symptoms. Oculogyric crises are common.	Generalized but often with axial hypotonia.	Progressive without treatment	l-DOPA response related to phenotype: response tends to be less good in those with complex encephalopathy. 5-HTP may be a useful adjunct
	*SGCE* (DYT11)	Childhood	Myoclonus. Neuropsychiatric manifestations	Cervical	Dystonia usually remains segmental	Myoclonus usually more prominent than dystonia. Often alcohol responsive
	*ATP1A3†* (DYT12)	Adolescence to adulthood	Parkinsonism	Rostocaudal gradient	Rapid onset then stabilizes	Onset in context of febrile illness or other stressor. No response to l-DOPA
	*PRKRA* (DYT16)	Childhood	Variable parkinsonism	Any body part	Generalization	No response to l-DOPA

Features described relate to typical cases and it must be borne in mind that there is often wide phenotypic variation between cases. † = clinical description restricted to rapid-onset dystonia-parkinsonism only (for alternating hemiplegia of childhood, see main text).

### The primary pure dystonias

Primary pure dystonia is usually defined as a syndrome in which dystonia is the only clinical feature (except for tremor of the arms or head and neck) without any evidence of neurodegeneration or any obvious secondary cause (e.g. trauma, autoimmune, post-infectious etc.).

#### TOR1A mutations (DYT1/Oppenheim’s disease)

In 1911, Oppenheim proposed the term ‘dystonia musculorum deformans’ to describe a syndrome in children with twisting or jerking movements, muscular spasm, and gait abnormalities that often progressed to a fixed postural deformity. The hereditary nature of the condition was subsequently recognized and, in 1990, the disease was linked to chromosome 9q32-34 and designated with the first dystonia locus (DYT1) (Kramer *et al.*, 1990). It was quickly realized that this locus was associated with a significant proportion of all childhood-onset dystonia, particularly in Ashkenazi Jews (Ozelius *et al.*, 1992; Kramer *et al.*, 1994). Seven years later the gene responsible for the condition was identified and named *TOR1A* (Ozelius *et al.*, 1997). The *TOR1A* gene comprises five exons and is widely expressed. In almost all cases of DTY1-related dystonia, the underlying genetic mutation is an inframe GAG deletion located in exon 5 of the gene, resulting in the loss of a single glutamic acid in the final protein product (torsin A). This mutation has been shown to be responsible for ∼80% of all primary, early-onset dystonia in Ashkenazi Jewish populations and up to 50% of primary, early-onset dystonia in non-Jewish populations (Bressman, 2006; Jamora *et al.*, 2006). Only two other missense variants (p.A288G and p.F205I) and one frameshift, 4-base pair deletion (c.934_937delAGAG) in this gene have since been reported to cause dystonia, though their pathogenicity is by no means certain (Kabakci *et al.*, 2004; Zirn *et al.*, 2008; Calakos *et al.*, 2010).

The penetrance of the GAG deletion is notably low, with current estimates somewhere within the region of 30–40% (Kramer *et al.*, 1994; Bressman, 2006). Moreover, even when the condition does manifest, the spectrum of severity of the symptoms is wide, ranging from mild focal dystonia to severe and disabling generalized dystonia. The exact mechanism underlying this variability remains unclear, but is presumed that other genetic or environmental modifiers must exist that influence the penetrance and presentation of the disease. To date, only one coding polymorphism in exon 4 of *TOR1A* (rs1801968), which encodes either aspartic acid (D) or histidine (H), has convincingly been shown to modify the risk of developing symptoms of dystonia. It appears that carrying the H allele in *trans* (that is, on the opposite allele from that harbouring the GAG deletion) may reduce the risk of developing symptoms of dystonia by 10-fold, to as little as 3% (Risch *et al.*, 2007; Kamm *et al.*, 2008*a*). Interestingly, in cellular models, the overexpression of *TOR1A* containing the H allele was sufficient by itself to induce formation of inclusions of torsin A in a manner similar to expression of GAG-deleted *TOR1A*, though at a much lower level (Kock *et al.*, 2006). It is thought that this may be the result of a disruption of protein interactions induced by the presence of the exposed histidine residue. However, expression of GAG-deleted *TOR1A* containing the H allele in *cis* actually reduced the formation of inclusions, implying that these two changes act to cancel each other out to some degree (Kock *et al.*, 2006). It has even been suggested that the GAG-deletion may need to be carried in conjunction with a D allele in *cis* to be penetrant (Risch *et al.*, 2007), though this has not yet been proven. Yet, although the effect of the 216H coding polymorphism appears to be robust (in *trans*, at least), its relatively low population frequency (∼12% in Europeans and less in other populations) means it cannot fully explain the reduced penetrance or variable expressivity seen in this condition; other factors clearly remain to be identified.

In terms of non-genetic factors that might affect the penetrance of TOR1A mutations, one small study has assessed exposure to perinatal adversity, childhood infections, general anaesthesia and trauma in manifesting and non-manifesting carriers as well as non-carriers from a series of 28 families with DYT1-related dystonia by means of a retrospective questionnaire (Martino *et al.*, 2012). Only self-reported perinatal adversity, in particular complications of vaginal delivery, showed a statistically significant (*P = *0.02) positive correlation with manifestation of the disease.

Clinically, DYT1-related dystonia typically presents in childhood with dystonic posturing of the foot or leg, though it may begin in any part of the body, and evolves to generalized dystonia with fixed deformities. As mentioned above, however, late-onset or much milder forms of the disorder are recognized (Jamora *et al.*, 2006). There is generally a family history consistent with autosomal dominant inheritance, albeit with reduced penetrance, but apparently sporadic early and late onset cases are also seen (Hjermind *et al.*, 2002).

The *TOR1A* gene encodes a protein torsin A, which is a member of the AAA+ family of ATPases and is believed to be involved in maintenance of both structural integrity and/or normal function of protein processing and trafficking. Torsin A is expressed throughout the CNS in humans, but is found at particularly high levels in the dopaminergic neurons of substantia nigra pars compacta, locus coeruleus, Purkinje cells, cerebellar dentate nucleus, basis pontis, thalamus, hippocampal formation, oculomotor nuclei and frontal cortex (Shashidharan *et al.*, 2000; Konakova *et al.*, 2001; Rostasy *et al.*, 2003). The exact mechanisms by which mutant torsin A causes dystonia are still unclear, but there is accumulating data to suggest that torsin A is important for cellular compartments and pathways, including the cytoskeleton, the nuclear envelope, the secretory pathway and the synaptic vesicle machinery (Goodchild and Dauer, 2005; Goodchild *et al.*, 2005; Hewett *et al.*, 2006, 2007; Henriksen *et al.*, 2009; Kakazu *et al.*, 2012).

#### THAP1 mutations (DYT6)

After the discovery of *TOR1A* and the subsequent uptake of genetic testing for mutations in this gene, it became clear that a second, distinct form of pure primary dystonia existed. Affected individuals, who were negative for *TOR1A* mutations, tended to have an older age at onset—in their adolescence or adulthood—and presented predominantly with cranio-cervical or focal dystonia affecting the upper limbs. In 1997, the DYT6 locus was assigned to chromosome 8p21-q22 by linkage analysis in two Mennonite families, but it was not until 2009 that the gene responsible for the condition was finally identified as *THAP1* (thanatos associated protein domain containing, apoptosis associated protein 1) (Almasy *et al.*, 1997; Fuchs *et al.*, 2009). Missense, nonsense and frameshift mutations, spread throughout most of the coding portion of the gene, have all been associated with disease. Mutations in this gene have been detected in genetically diverse populations throughout the world and, unlike *TOR1A*, do not seem to be especially prevalent in any one particular population. Inheritance is autosomal dominant with a reduced penetrance. To date, penetrance has only been measured in Amish–Mennonite families, where it appears to be ∼60%, but this may not be true of all populations or mutations (Saunders-Pullman *et al.*, 2007). Most mutations are found in the heterozygous state, but homozygotes are described (Fuchs *et al.*, 2009; Houlden *et al.*, 2010).

The clinical spectrum of *THAP1* mutations is wide. Presentation with oromandibular, cranio-cervical or laryngeal dystonia is common, but presentations with focal dystonia of the limbs, segmental or generalized dystonia are all described in the literature. In a recent analysis by Xiromerisiou *et al.* (2012) of 100 patients reported to carry *THAP1* mutations, the mean age at onset of dystonia was 24 years, 60% of patients were females and the distribution was generalized in 37%, segmental in 30%, multifocal in 6% and focal in 27%.

The THAP1 protein is an atypical zinc finger protein characterized by an N-terminal THAP domain, a proline-rich region and a C-terminal nuclear localization domain (Roussigne *et al.*, 2003). The THAP domain is conserved in both vertebrates and invertebrates (Clouaire *et al.*, 2005). It has DNA binding properties and is thought to be involved in the regulation of transcription, either on its own or in concert with other proteins. Thus, one possible mechanism by which mutations in *THAP1* might cause dystonia is by dysregulated transcription of key genes. THAP1 protein is also known to interact with the prostate apoptosis response protein 4 (PAR-4), which is a transcription regulator involved in apoptosis (Roussigne *et al.*, 2003). Under conditions of cellular stress or toxicity, this protein is rapidly upregulated and it has been linked to neurodegeneration in a number of disease models (Guo *et al.*, 1998; Duan *et al.*, 1999; Pedersen *et al.*, 2000). One intriguing finding has been that THAP1 may regulate transcription of *TOR1A* by binding to one of two sites in its promoter region (Gavarini *et al.*, 2010; Kaiser *et al.*, 2010). Mutations in *THAP1* have been shown to disrupt binding to the *TOR1A* promoter and decrease *TOR1A*-driven luciferase expression, thus suggesting that under some conditions *TOR1A* is negatively regulated by THAP1 protein and that mutations in *THAP1* may lead to abnormally high levels of torsin A (Kaiser *et al.*, 2010). Although these data come from experimental cell lines and thus are of uncertain physiological significance, this does at least raise the possibility that the disease mechanisms in DYT1 and DYT6 dystonia may be linked. However, if this is the case, the question arises as to how to link this mechanistically with the prevailing idea that the GAG-deletion in *TOR1A* leads to a loss of function of the protein (Goodchild *et al.*, 2005). One possibility is that *TOR1A* expression must be maintained within a set range in relation to its binding partners and that dysfunction may result from either increased expression or loss of function (Bragg *et al.*, 2011). Indeed, transgenic mice overexpressing wild-type *TOR1A* have been shown to display evidence of neurohistological, neurochemical and behavioural abnormalities, which may provide some support for this hypothesis (Grundmann *et al.*, 2007).

#### ANO3 mutations (DYT23)

Recently, our laboratory employed a combination of exome sequencing and linkage analysis in moderately-sized, three generation Caucasian kindred with autosomal dominant cranio-cervical dystonia to identify a mutation in *ANO3* (p.Arg494Trp) that segregated with the disease in the family (Charlesworth *et al.*, 2012). Subsequent screening of this gene in 188 probands with cervical dystonia revealed five further novel variants that were not found in any databases of sequence variation, scattered throughout the gene, including one variant in its 5’ UTR. Segregation analysis was possible for two of these additional variants (p.Trp490Cys and p.Ser685Gly), which were seen to segregate with disease. The expression pattern of the gene was supportive of its role in dystonia, with expression being several folds higher in the striatum than anywhere else (Charlesworth *et al.*, 2012).

Clinically, patients with mutations in this gene exhibited focal or segmental dystonia, variably affecting the cranio-cervical, laryngeal or brachial regions. There was often dystonic tremor with a jerky quality affecting the head, voice or upper limbs. The age at onset ranged from the very early childhood to 40 years old. Interestingly, despite prolonged follow-up and a gradual increase in severity, the dystonia was never seen to generalize in any affected individual, remaining confined to the head, neck and upper limbs. Some individuals manifested upper limb tremor alone and had been misclassified as essential tremor.

*ANO3* encodes a protein called anoctamin 3, about which little is yet known. It belongs to a family of genes (*ANO1–10*) that appear to be closely related in sequence and topology, but with distinct expression patterns. Several of these genes have already been linked to various diseases, suggesting they play an important role within their tissue types (Duran and Hartzell, 2011). *ANO1* and *ANO2*, the best studied members of the family, encode proteins that function as Ca^2+^-activated chloride channels and it is possible anoctamin 3 may function in the same manner (Caputo *et al.*, 2008). Certainly, hydropathy analysis suggests a similar topology, with eight hydrophobic helices that are likely to be transmembrane domains and cytosolic N- and C-termini, but more recent work has suggested that anoctamin 3 may in fact be targeted to the endoplasmic reticulum, rather than the cell surface, like anoctamins 1 and 2 (Duran *et al.*, 2012). Moreover, patient fibroblasts habouring the p.Trp490Cys mutation showed evidence of a potential defect in endoplasmic reticulum related Ca^2+^ handling. It is believed that Ca^2+^-activated chloride channels have a role to play in the modulation of neuronal excitability (Hartzell *et al.*, 2005; Huang *et al.*, 2012) and, in view of high expression of *ANO3* in the striatum, it is possible that mutations in this gene lead to abnormal excitability of striatal neurons, which manifests itself clinically in unwanted dystonic movements, though further functional work will be required to test this hypothesis.

#### GNAL mutations

At the same time, mutations in another gene, *GNAL*, were identified as further cause of familial primary pure dystonia (Fuchs *et al.*, 2012). Using whole exome sequencing and linkage in two unrelated families, two mutations in *GNAL* were initially identified, a nonsense mutation (p.Ser293*) resulting in a premature stop codon in one family and a missense mutation (p.Val137Met) in the other. Screening of 39 additional families identified six further novel mutations, with segregation confirmed in all families for which DNA from additional affected individuals was available.

Clinically, of the 28 individuals with dystonia resulting from a mutation in *GNAL*, 82% had onset in the region of the neck and 93% had cervical involvement when examined for the study. However, progression to other sites had occurred in at least half of those affected and generalized dystonia was seen in ∼10% of cases. Thus, the clinical phenotype in *GNAL*-related dystonia appears to be not dissimilar to that caused by mutations in *THAP1*. Though the authors noted that onset in *GNAL* mutations was never brachial, this may just be a chance finding given the small number of cases identified so far, rather than a true distinguishing factor.

*GNAL* encodes Gα_olf_, the alpha subunit of triheteromeric G protein G_olf_, which is involved in dopamine (D1) signalling. Experimental evidence exists to show that Gα_olf_ is mostly responsible for the coupling of D1 receptors to adenylyl cyclase in striatal neurons and that Gα_olf_ is required for D1-mediated behaviour and biochemical effects (Herve *et al.*, 1993; Zhuang *et al.*, 2000; Corvol *et al.*, 2001). Because D1 dopamine receptors have a known role in mediating locomotor activity, the link between *GNAL* and dystonia is biologically plausible. The gene is located on the short arm of chromosome 18 in a region that has been linked not only to DYT7 dystonia (though the original DYT7 family do not appear to carry a mutation in *GNAL*), but also to bipolar disorder, schizophrenia and attention deficit hyperactivity disorder (Leube *et al.*, 1997*a*; Berrettini, 2000; Segurado *et al.*, 2003; Laurin *et al.*, 2008; Winter *et al.*, 2012). Interestingly, homozygous knockout mice for this gene are hyposmic and display hyperactive behaviours (Belluscio *et al.*, 1998).

#### CIZ1 mutations

In early 2012, Xiao *et al.* (2012) reported that they had used a combination of linkage analysis and whole exome sequencing to identify a mutation in *CIZ1* (p.Ser264Gly) as the likely causal variant in a large Caucasian kindred with primary cervical dystonia inherited as an autosomal dominant trait. Those affected in the family exhibited focal cervical dystonia, occasionally with mild tremor, having its onset in early adulthood to late midlife (18–66 years of age). However, affectation status was not always clear cut: five family members were said to have ‘definite’ cervical dystonia, whereas five family members were said to have ‘possible’ cervical dystonia, making linkage analysis difficult (Uitti and Maraganore, 1993).

*CIZ1* encodes CDKN1A interacting zinc finger protein 1, a p21^Cip1/Waf1^-interacting zinc finger protein expressed in the brain and involved in DNA synthesis and cell-cycle control. Functional work suggests that the p.Ser264Gly mutation may alter splicing of the gene and normal subnuclear localization of CIZ1 protein (Xiao *et al.*, 2012). Screening in a cohort of patients with adult-onset dystonia identified two additional missense mutations in three individuals (p.Pro47Ser and p.Arg672Met), all with focal cervical dystonia developing in mid-to-late life.

Some researchers feel it may be too early to place full confidence in the association of mutations in *CIZ1* and focal cervical dystonia. Firstly, databases of normal sequence variation reveal that the *CIZ1* gene contains a large number of missense variants in supposedly healthy individuals (a third of which are predicted to be pathogenic) and, in the study by Xiao *et al.* (2012) an equal number of novel missense variants were found in controls as were found in patients. Secondly, and more importantly, segregation was not demonstrated in a second independent family for any of the purported mutations. Thirdly, there is some question regarding the quality of the exome data used in this study: coverage was only >20 reads for ∼60% of the exome and the number of false-positive variants appears unusually high (Klein *et al.*, 2012). Screening of this gene in further cohorts of cervical dystonia cases from various populations will be required to decide if these reservations are warranted or not.

#### TUBB4A mutations (DYT 4)

DYT4 was first described in 1985 by forensic psychiatrist Neville Parker in a large family with third decade onset of autosomal dominantly inherited ‘whispering dysphonia’ and generalized dystonia (Parker, 1985). Over 25 affected individuals have been reported, typically presenting with a laryngeal dysphonia progressing to a generalized dystonia and a peculiar ‘hobby horse’ gait (Wilcox *et al.*, 2011). Alcohol responsiveness was not uncommon, leading to severe alcohol abuse in some DYT4 patients (Wilcox *et al.*, 2011). The family is descended from an affected male who was born in 1801 in the small rural coastal town of Heacham in Norfolk and, to date, no other kindred has been described worldwide with a similar phenotype.

Using a combination of linkage analysis and exome sequencing, a mutation in the gene *TUBB4A* (p.Arg2Gly) has recently been identified as causal in the DYT4 kindred by two groups independently (Hersheson *et al.*, 2012; Lohmann *et al.*, 2012). *TUBB4A* encodes β-tubulin 4 a, a constituent of microtubules, and the mutation results in an arginine to glycine amino acid substitution in the key, highly conserved autoregulatory MREI (methionine–arginine–glutamic acid–isoleucine) domain of the protein. The gene is expressed throughout the brain, but at the highest levels in the cerebellum, which has been linked to the pathogenesis of dystonia (Hersheson *et al.*, 2012). The MREI tetrapeptide sequence at the start of the N-terminal domain is known to be necessary for the autoregulation of the β-tubulin messenger RNA transcript and separate *in vitro* studies using site-directed mutagenesis have previously demonstrated that the p.Arg2Gly mutation abrogates this autoregulatory ability (Yen *et al.*, 1988*a*, *b*). One further possibly pathogenic variant (p.Ala271Thr) was detected during the screening of a cohort of 394 unrelated dystonia patients: the individual concerned exhibited spasmodic dysphonia with oromandibular dystonia and dyskinesia with an age at onset of 60. Her mother had been similarly affected, but was now deceased so that segregation analysis was not possible, meaning the pathogenicity of the variant is uncertain (Lohmann *et al.*, 2012).

### The paroxysmal dystonias

The paroxysmal dystonias are characterized, in theory at least, by episodes of dystonia or other dyskinesia separated periods of neurological normality. In practice, there may be other associated neurological features besides the movement disorder and, especially in the case of SLC2A1-related disease, both the movement disorder and the other associated neurological symptoms may become relatively fixed with time.

#### MR-1 mutations (DYT8)

Symptoms of paroxysmal non-kinesigenic dyskinesia (PNKD) typically begin in childhood or adolescence and include dystonic and choreatic dyskinesias or ballistic movements, lasting from minutes to hours. There are often premonitory symptoms of an impending attack (paraesthesia or tension in the affected area) and sleep can prevent or abort attacks. The frequency of attacks varies between individuals, ranging from daily attacks to only a few in a lifetime. Typically, they are precipitated by alcohol, caffeine or stress, but less often by exercise, fatigue or cold (Bruno *et al.*, 2007). Neurological examination is normal between attacks. Treatment is with carbamazepine or benezodiazepines, particularly clonazepam.

The condition results from mutations in the myofribillogenesis regulator gene (*MR-1*, also known as *PNKD*) and is inherited as an autosomal dominant trait. Only three mutations have been described to date, all clustered in the N-terminus of the protein: p.Ala7Val, p.Ala9Val and p.Ala33Pro (Lee *et al.*, 2004; Rainier *et al.*, 2004; Chen *et al.*, 2005; Djarmati *et al.*, 2005; Hempelmann *et al.*, 2006; Ghezzi *et al.*, 2009). Despite this, haplotype analysis has failed to reveal a common founder, suggesting that these represent independent mutational events (Chen *et al.*, 2005). It is currently believed that this region of the protein may represent a mitochondrial targeting sequence (Ghezzi *et al.*, 2009). Mutations may act by disrupting protein processing *in vivo*, a hypothesis that is supported by evidence from transgenic mice (Shen *et al.*, 2011). The function of the *MR-1* protein is not fully understood, but it shows close homology to glyoxalase hydroxyacylglutathione hydrolase, which is known to detoxify methylglyoxal, a compound found in coffee and alcohol and a by-product of glycolysis, thus providing a link to the standard triggers (Lee *et al.*, 2004).

#### PRRT2 mutations (DYT10)

Mutations in *PRRT2* cause paroxysmal kinesigenic dyskinesia (PKD), which has an estimated prevalence 1 in 150 000 individuals (Bruno *et al.*, 2004). Affected individuals have short (seconds to minutes) and frequent (up to 100 times per day) attacks of dystonic or choreiform movements, precipitated by sudden movements or startle. As with paroxysmal non-kinesigenic dyskinesia, there is often warning of an impending attack—a so-called ‘aura’—consisting of numbness or paraesthesia in the affected body part. The attacks usually begin in childhood and are highly responsive to anticonvulsant therapy, such as carbamazepine (Silveira-Moriyama *et al.*, 2013). Reports of what was, in reterospect, probably this disorder have appeared in the literature from as early as 1892 and the condition was designated dystonia 10 in 1998 (Smith and Heersema, 1941; Muller *et al.*, 1998). However, the discovery of the gene underlying the condition, *PRRT2*, would wait until exome sequencing approaches could be applied to the problem (Chen *et al.*, 2011; Wang *et al.*, 2011).

Most mutations are truncating and by far the most common of these is the c.649dupC mutation, but missense variants (possibly with reduced penetrance) have also been described (Cao *et al.*, 2012; Friedman *et al.*, 2012*a*; Hedera *et al.*, 2012; Steinlein *et al.*, 2012). The PRRT2 protein is highly expressed in the CNS and is probably localized to the synapse (Lee *et al.*, 2012). Using yeast 2-hybrid screening, it has been shown to interact with synaptosomal protein 25 (SNAP25), suggesting a role in the fusion of the synaptic vesicles to the plasma membrane (Stelzl *et al.*, 2005). Both PRRT2 and SNAP25 are highly expressed in the basal ganglia and disrupted neurotransmitter release has been suggested as a possible pathogenic mechanism in *PRRT2* mutations. Truncating mutations produce a protein lacking the transmembrane domain, and are thought to result in altered subcellular localization (Chen *et al.*, 2011). Missense mutations may cause loss of function or act in a dominant-negative manner (Wang *et al.*, 2011; Li *et al.*, 2012).

*PRRT2-*related disease is notable for its varied presentation, differing not merely between individuals carrying different mutations, but also between individuals carrying the same mutation and even between affected members within the same family (Silveira-Moriyama *et al.*, 2013). As well as the classical paroxysmal kinesigenic dyskinesia phenotype described above, infantile convulsions with choreoathetosis (ICCA) with or without paroxysmal kinesigenic dyskinesia, benign familial infantile seizures, episodic ataxia, hemiplegic migraine and even benign paroxysmal torticollis of infancy all appear to be possible manifestations of mutations in this gene (Dale *et al.*, 2012; Gardiner *et al.*, 2012; Heron *et al.*, 2012; Lee *et al.*, 2012). The common thread would seem to be the paroxysmal nature of all *PRRT2*-related disorders.

Finally, it would appear that the family used to define DYT19 also carry the common c.649dup mutation in *PRRT2*, suggesting that the initial linkage was incorrect (Gardiner *et al.*, 2012).

#### SLC2A1 mutations (DYT18)

Dominantly inherited mutations in the *SLC2A1* gene are now known to be the cause of paroxysmal exercise-induced dyskinesia (PED) (Suls *et al.*, 2008; Weber *et al.*, 2008). In fact, *SLC2A1* mutations cause a wide spectrum of disease, resulting from a deficiency of the encoded glucose transporter type 1, which is still commonly referred to as GLUT1 despite the change in the recommended gene name. GLUT1 is the principal glucose transporter in the brain and it has generally been assumed that neuronal dysfunction arises from energy failure. Under this assumption, the prevalence of movement disorders was thought to reflect the heightened sensitivity of the basal ganglia to energy deficits (Perez-Duenas *et al.*, 2009). However, recent studies in a mouse model of GLUT1-deficiency, which recaptures many of the features of the disease, demonstrated the preservation of aerobic metabolism, such that mechanisms other than impaired aerobic glycolysis must be responsible for the manifestations of the disorder (Marin-Valencia *et al.*, 2012). Possible culprits include reduced transfer of lactate generated through anaerobic glycolysis from the astrocyte into the neuron and reduced anaerobic ATP formation in the astrocyte having a negative impact on glutamate-glutamine recycling, which, if proven, would place the emphasis on glial dysfunction in the pathogenesis of GLUT1-deficiency (Magistretti and Pellerin, 1996; Marin-Valencia *et al.*, 2012).

At its most severe, GLUT1 deficiency can present with early onset psychomotor delay, drug resistant epilepsy, acquired microcephaly and other signs of widespread neurological dysfunction, such as spasticity, ataxia, tremor, dystonia, choreoathetosis and ballism, which may all be paroxysmal, static or static with paroxysmal worsening. From this extreme, there exists an entire spectrum of decreasing disease severity, ranging through developmental delay and movement disorders without epilepsy, right down to the mildest cases, in which only minimal phenotypic abnormalities are detectable (Brockmann, 2009; Leen *et al.*, 2010). There can be considerable heterogeneity of clinical presentation even within the same family (Leen *et al.*, 2010).

Paroxysmal exercise-induced dyskinesia is, therefore, probably best regarded as just one possible manifestation of the complex and variable disorder that is GLUT1-deficiency syndrome. It is characterized by attacks of combined chorea, athetosis, and dystonia precipitated by exercise—particularly brisk walking or running—that usually begin in childhood (Suls *et al.*, 2008; Weber *et al.*, 2008). The legs and feet are by far the most commonly affected body parts during attacks, which generally last from a few minutes to an hour (Bruno *et al.*, 2004). Unlike paroxysmal kinesigenic dyskinesia or paroxysmal non-kinesigenic dyskinesia, affected individuals do not report aura-like symptoms before an attack. The symptoms tend to improve with intravenously administered glucose and with permanent ketogenic diet, though they can become static with time (Weber *et al.*, 2008). Because paroxysmal exercise-induced dyskinesia is simply a manifestation of GLUT1 deficiency, it may occur alone in minimally affected patients or may be accompanied by various other manifestations of the disease, such as epilepsy, ataxia, haemolytic anaemia or spastic paraplegia. No doubt this wide variation in clinical presentation contributed to the erroneous assignation of DYT9 (paroxysmal choreoathetosis with spasticity) and DYT18 (PED with or without epilepsy) as two separate conditions; it is now known that the causative gene in both cases was, in fact, *SLC2A1* (Weber *et al.*, 2011).

Given the variable expressivity of *SLC2A1* mutations, researchers have sought correlations between genotype and phenotype. It has been suggested that multiple exon deletion is associated with the early-onset, more severe form of the disorder, whereas missense mutations are associated with less severe cognitive deficits and a lower rate of movement disorders (Leen *et al.*, 2010).

### The dystonia-plus syndromes

The dystonia-plus syndromes represent a heterogeneous group of diseases, where dystonia is accompanied by other neurological features but is not part of a complex neurodegenerative disorder.

#### SGCE mutations (DYT11)

Mutations in *SGCE* cause myoclonus-dystonia, the commonest of the ‘dystonia-plus’ syndromes with a prevalence of about 2 per million in Europe (Asmus *et al.*, 2007). The condition is inherited in an autosomal dominant fashion but with a significant role played by imprinting (see below). It usually begins in childhood, with a mean age of onset of ∼6 years, and is characterized by brief lightning-like myoclonic jerks, most often affecting the neck, trunk and upper limbs, in combination with focal or segmental dystonia in around two-thirds of patients (Asmus and Gasser, 2004). Myoclonus usually dominants the clinical picture and can be reliably provoked by complex motor activities, such as writing or copying a picture. There is often a dramatic improvement after alcohol, but this feature is not specific to the condition and is not present in all cases (Nardocci, 2011).

The *SGCE* gene encodes the 438 amino acid protein ε-sarcoglycan, which contains a single transmembrane domain. Despite its ubiquitous expression pattern and close homology to α-sarcoglycan (68%), mutations in *SGCE* do not lead to any detectable deficits in peripheral nerve or muscle function. Over 50 different mutations have been reported as causing myoclonus-dystonia, most of them located within the portion of the gene encoding the extracellular domain of the protein (Kinugawa *et al.*, 2009). It is thought that mutations lead to mislocalization of the protein from the plasma membrane to the endoplasmic reticulum and to the promotion of its degradation by the proteasome (Esapa *et al.*, 2007). Interestingly, in humans, transcription occurs almost exclusively from the paternal allele, whereas transcription from the maternal allele is silenced by promoter methylation (Grabowski *et al.*, 2003). Because of this fact, 95% of those inheriting an *SGCE* mutation from their mother will not manifest any symptoms of the disease.

In patients exhibiting alcohol responsiveness, GABAergic drugs such as clonazepam or gamma-hydroxybutyrate often produce a pronounced but temporary improvement in the condition. However, their short duration of action can lead to overuse and addiction and this danger is compounded by apparently higher rates of psychiatric comorbidity—particularly obsessive–compulsive disorder, anxiety and alcohol dependency—in *SGCE* mutation carriers (Hess *et al.*, 2007; Peall *et al.*, 2013). The myoclonus seen in this condition is subcortical and does not respond to agents used to treat cortical myoclonus, such as valproate, levetiractem or piracetam (Roze *et al.*, 2008). Levodopa responsiveness (of both the myoclonic and dystonic elements of the condition) has been reported in two cases and some have suggested a trial of levodopa is warranted (Luciano *et al.*, 2009).

#### ATP1A3 mutations (DYT12)

Mutations in *ATP1A3* are primarily associated with rapid onset dystonia-parkinsonism. Familial cases show autosomal dominant inheritance with reduced penetrance (∼90%), but *de novo* mutations are common so that the absence of a family history should not preclude consideration of the condition (de Carvalho Aguiar *et al.*, 2004). Most mutations are missense, affecting highly conserved transmembrane or N-terminus domains. The mechanism by which such mutations actually cause rapid onset dystonia parkinsonism is not fully understood. *ATP1A3* encodes the catalytic unit of a sodium pump that uses ATP hydrolysis to exchange Na^+^ and K^+^ across the cell membrane, thus maintaining the ionic gradients important for electrical excitability, neurotransmitter transport, volume regulation and other key cellular functions. Studies using cells transfected with mutant ATP1A3 have suggested mutations may reduce affinity for Na^+^ and that the resultant dysfunction in ion transport may impair cell viability, but the relative importance of these mechanisms to the actual pathophysiology of rapid onset dystonia-parkinsonism remains uncertain (Rodacker *et al.*, 2006; Blanco-Arias *et al.*, 2009).

Clinically, the condition is characterized by the onset, over minutes to days, of dystonia and parkinsonism, usually in the context of specific physical or psychological stressors, particularly febrile illness. After this period of rapid development of symptoms, the condition usually stabilizes and continued progression has only been reported once (Brashear *et al.*, 2007). There is often a rostrocaudal gradient of symptoms. Despite clinical parkinsonism, dopamine transporter PET and single-photon emission computed tomography studies reveal normal dopamine uptake (Brashear *et al.*, 1999; Zanotti-Fregonara *et al.*, 2008) and, unfortunately, patients with rapid-onset dystonia parkinsonism do not respond to levodopa or pallidal deep brain stimulation (Deutschlander *et al.*, 2005; Kamm *et al.*, 2008*b*).

Interestingly, *de novo* heterozygous mutations in *ATP1A3* also appear to underlie a rare neuropaediatric condition termed alternating hemiplegia of childhood (AHC) (Heinzen *et al.*, 2012; Rosewich *et al.*, 2012). Alternating hemiplegia of childhood is characterized by the onset before the age of 18 months of paroxysmal neurological events, such as hemiplegia alternating in laterality, quadriplegia, dystonic spells, oculomotor abnormalities and autonomic dysfunction, all of which abate during sleep. Non-paroxysmal manifestations develop after a few months or years of the disease, comprising developmental delay, intellectual disability of variable degree, ataxia, dysarthria, choreoathetosis, and, in some patients, pyramidal tract signs. In one study, a mutation in *ATP1A3* was demonstrated in all 24 patients with alternating hemiplegia of childhood (Rosewich *et al.*, 2012). A second study found *ATP1A3* mutations is just under three-quarters of their cases (Heinzen *et al.*, 2012). One mutation, p.Asp801Asn, was found 30–40% of all cases, suggesting it is a mutational hot spot.

#### PRKRA mutations (DYT16)

Mutations in the gene *PRKRA* appear to be a rare cause of autosomal recessive dystonia-parkinsonism. Camargos *et al.* (2008) identified two apparently unrelated consanguineous Brazilian families with a total of six individuals exhibiting young-onset, generalized dystonia or dystonia-parkinsonism. Autozygosity mapping revealed a shared segment of homozygosity segregating with the disease and subsequent mutational screening of the region identified a homozygous missense mutation (p.Pro222Lys) in all six individuals (Camargos *et al.*, 2008). *PRKRA* encodes protein kinase, interferon-inducible double-stranded RNA-dependent activator, which, in response to extracellular stress activates the latent protein kinase PKR, a protein involved in signal transduction, cell differentiation, cell proliferation, antiviral response and apoptosis (Patel *et al.*, 2000). The Pro222Lys mutation alters a conserved amino acid between the second and third RNA-binding motifs, but the manner by which it causes disease is remains elusive. As with patients carrying mutations in *ATP1A3*, response to standard medical therapy was minimal or absent.

### DOPA-responsive dystonia

**Figure 2:**
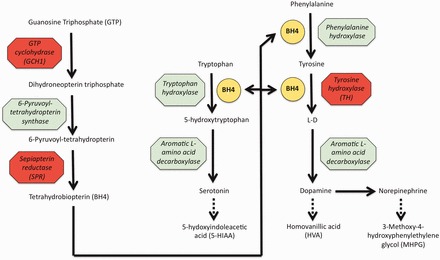
Schematic illustration of the pathways for the synthesis of catecholamines and serotonin. Breakdown products are indicated by broken arrows. Enzymes in which defects cause DOPA-responsive dystonia are shown in red with the gene symbol in brackets. Tetrahydrobiopterin is a key cofactor in some enzymatic reactions.

Technically, the DOPA-responsive dystonias fall within the category of the ‘dystonia-plus’ syndromes, but they have been separated out in this discussion because they are bound together aetiologically by their connection to the endogenous pathway for the synthesis of dopamine, which accounts for their shared clinic characteristic of an improvement in symptoms in response to treatment with oral l-DOPA. To date, three genes have been convincingly shown to cause DOPA-responsive dystonia: *GCH1* (GTP cyclohydrolase 1), *TH* (tyrosine hydroxylase) and *SPR* (sepiapterin reductase) (Ichinose *et al.*, 1994; Brautigam *et al.*, 1998; Bonafe *et al.*, 2001). All three genes encode enzymes required for the biosynthesis of dopamine (Fig. 2).

#### GCH1 mutations (DYT5a/Segawa’s disease)

Mutations in this gene account for ∼60–80% of autosomal dominant DOPA-responsive dystonia (Furukawa, 2004). Penetrance is incomplete and lower for males (∼40–50%) than females (∼80%) (Wider *et al.*, 2008). *GCH1* encodes the enzyme GTP cyclohydrolase 1, which catalyzes the ﬁrst step in the reaction of tetrahydrobiopterin neo-synthesis from GTP. Tetrahydrobiopterin is an essential cofactor for tyrosine hydroxylase, the rate-limiting enzyme for catecholamine synthesis, as well as for tryptophan hydroxylase and phenylalanine hydroxylase, which are involved in the production of serotonin. *GCH1* mutations act in a dominant negative manner, with measurable enzyme activity usually being <20% of normal, resulting in a relative deficiency of dopamine and serotonin (Ichinose and Nagatsu, 1997; Hwu *et al.*, 2000). Diagnosis can be confirmed on the basis of reduced CSF levels of total biopterin (of which tetrahydrobiopterin is the main component) and neopterin (a product of the reaction involving GCH1) (Table 6) or an abnormal phenylalanine loading test (Bandmann *et al.*, 2003; Furukawa, 2003).

**Table 6:** CSF neurotransmitter metabolite profiles in DOPA-responisve dystonia secondary to *GCH1*, *TH* and *SPR* mutations

CSF metabolite	GCH1	TH	SPR
Biopterin	Decreased	Normal	Normal to slightly raised
Neopterin	Decreased	Normal	Increased
Homovanillic acid	Normal to mildly decreased	Decreased	Very low
5-HIAA	Normal to mildly decreased	Normal	Very low
MHPG	Normal to mildy decreased	Decreased	Decreased

5-HIAA = 5-hydroxyindoleactetic acid; MHPG = 3-methoxy-4-hydroxy-phenylethylene glycol.

Clinical presentation is typically with lower limb dystonia and gait disturbance in the first decade of life, which occasionally leads to misdiagnosis as cerebral palsy or spastic paraparesis (Nygaard *et al.*, 1990). Diurnal fluctuation in the severity of the dystonia is common, though this may abate with age. With time there is usually gradual generalization, and parkinsonism and dystonic tremor are possible complications of the condition (Segawa *et al.*, 2003; Cobb *et al.*, 2009). In a subgroup of patients, the dystonia is mild, progresses only very slowly and may require no treatment (Trender-Gerhard *et al.*, 2009). Infrequently, patients may present later in life with a dystonic tremor or akinetic-rigid parkinsonism (Tassin *et al.*, 2000; Trender-Gerhard *et al.*, 2009). There is some evidence that subtle neuropsychiatric features may be associated with mutations in this gene. In one recent study of 18 patients, major depressive and sleep disorders occurred in about half of those over 20 years of age, whereas obsessive–compulsive disorder was found in 25% of cases (Van Hove *et al.*, 2006). In terms of treatment, the response even to low doses of l-DOPA is generally excellent (70–100% improvement in clinical symptoms), sustained and is not generally associated with the late-onset dyskinesias that often accompany prolonged use of l-DOPA in other conditions, such as Parkinson’s disease (Clot *et al.*, 2009).

It should be noted that a previously-proposed novel form of DOPA-responsive dystonia, DYT14, is now known to be synonymous with DYT5a. Misclassification of one patient from the ‘DYT14’ family had led to incorrect locus assignment on the basis of erroneous linkage. After removal of this patient, the linkage peak now included the *GCH1* gene, which was found to habour a causative deletion (Wider *et al.*, 2008).

Finally, autosomal recessive mutations in *GCH1* have also been reported, resulting in no detectable enzyme activity in the liver. As might be expected the phenotype is generally distinct and much more severe, with complex neurological dysfunction, including developmental delay, spasticity, seizures and physiological hyperphenylalaninaemia, which is generally picked up on routine screening of newborn infants (Ichinose *et al.*, 1995). However, atypical presentations have been described with DOPA-responsive dystonia (Hwu *et al.*, 1999), with a neonatal DOPA-responsive extrapyramidal syndrome (Nardocci *et al.*, 2003) or without hyperphenylaninaemia (Horvath *et al.*, 2008; Opladen *et al.*, 2011).

#### TH mutations (DYT5b)

Rarely, DOPA-responsive dystonia can also be inherited as an autosomal recessive disorder associated with mutations in the gene encoding tyrosine hydroxylase itself (Ludecke *et al.*, 1996). It is sometimes referred to as DYT5b. Only ∼40 cases have been reported worldwide but with over 30 different pathogenic mutations, scattered throughout the length of the gene, including its promoter regions (Willemsen *et al.*, 2010).

As shown in Fig. 2, tyrosine hydroxylase is involved in the conversion of phenylalanine to dopamine, but it is also the rate-limiting enzyme in the synthesis of other catecholamines, such as norepinephrine and epinephrine. The enzyme thus plays a key role in normal functioning of both dopaminergic and noradrenergic neuronal populations and, indeed, complete loss of tyrosine hydroxylase activity appears to be lethal in knock-out mice (Zhou *et al.*, 1995). Patients with biallelic mutations in this gene tend to present with a more severe disease than is seen in those carrying heterozygous *GCH1* mutations, which presumably reflects the more profound deficiency of dopamine and norephinephrine. Two major phenotypes can be discerned: (i) a progressive hypokinetic-rigid syndrome with dystonia beginning in the first year of life and significant improvement in response to l-DOPA; and (ii) a more complex encephalopathy, associated with variable degrees of hypokinesia, bradykinesia, hypotonia, dystonia, myoclonus, ptosis and eye movement abnormalities that has its onset in the first few months of life, is often poorly responsive to l-DOPA and carries a far worse long term prognosis (Clot *et al.*, 2009; Willemsen *et al.*, 2010). In the latter subtype, autonomic functions are often disturbed leading to so-called ‘lethargy-irritability crises’, consisting of excessive drooling, sweating, body temperature instability and marked periods of ‘pyrexia of unknown origin’ (Willemsen *et al.*, 2010).

In keeping with the underlying biochemical defects, CSF levels of homovanillic acid and 3-methoxy-4-hydroxyphenylethylene glycol (MHPG), the breakdown products of dopamine and norepinephrine, respectively, are found to be low. CSF levels of biopterin and 5-hydroxyindoleacetic acid are normal, however, reflecting the fact that the synthesis of tetrahydrobiopterin and serotonin is unaffected by mutations in the tyrosine hydroxylase gene (Table 6 and Fig. 2).

#### SPR mutations

Mutations in the gene *SPR* result in a second form of autosomal recessive DOPA-responsive dystonia. Deficiency of the encoded enzyme, sepiapterin reductase, results in neurologic deterioration due to severe catecholamine and serotonin deficiencies in the CNS, caused by the defect in tetrahydrobiopterin synthesis. As well as being a cofactor for tyrosine hydroxylase, tetrahydrobiopterin is also a required cofactor for phenylalanine hydroxylase, but patients with sepiapterin reductase deficiency do not exhibit overt hyperphenylalaninaemia as is seen in other autosomal recessive disorders of tetrahydrobiopterin synthesis as alternative enzymes are able to replace sepiapterin reductase in peripheral tissues and can thus compensate to some degree. In the brain, however, the low activity of dihydrofolate reductase means that an alternate pathway for tetrahydrobiopterin synthesis cannot complete and an intermediate, dihydrobiopterin, accumulates instead (Blau *et al.*, 2001). The accumulation of dihydrobiopterin is probably of pathological significance since it is a competitive inhibitor of both tyrosine and tryptophan hydroxylase, thus further compounding the deficits in catecholamine and serotonin production caused by the subnormal tetrahydrobiopterin concentrations. Furthemore, by displacing prebound tetrahydrobiopterin, it has been suggested that dihydrobiopterin may uncouple nitric oxide synthase and cause the release of potentially apoptotic levels of superoxide and peroxynitrite, resulting in neuronal cell death (Blau *et al.*, 2001; Jones *et al.*, 2001).

The complex biochemical abnormalities described above are reflected in the CSF metabolite measurements, which show severe decreases in homovanillic acid and 5-hydroxyindoleacetic acid in the context of a normal neopterin and a normal or slightly raised total biopterin (Table 6). The neurological phenotype of SPR deficiency is quite variable, with milder cases being particularly susceptible to misdiagnosis as cerebral palsy (Friedman *et al.*, 2012*b*). Most affected individuals will display motor and speech delay, axial hypotonia, dystonia, weakness and oculogyric crises with diurnal variation and sleep benefit, whereas dysarthria, parkinsonian features, hyperreflexia, behavioural abnormalities, mental retardation, and autonomic signs are not uncommon accompaniments (Bonafe *et al.*, 2001; Friedman *et al.*, 2012*b*). Many patients will show considerable benefit from treatment with low-dose l-DOPA, with most improvement being observed in motor and sleep symptoms. Significant cognitive problems may remain, however, and some patients have shown further benefit from the addition of 5-hydroxytryptophan, a serotonin precursor, leading to the suggestion that it should be trialled in all patients without complete symptom resolution on l-DOPA alone (Friedman *et al.*, 2012*b*).

### Mechanistic insights from the monogenic primary dystonias

As with other disorders, it is believed that an understanding of the genetic architecture of dystonia offers that best starting point for the complex task of unravelling the molecular pathophysiology of the disorder. Identification of genes involved in rarer, Mendelian forms of dystonia, along with a knowledge of their cellular function, allows us to identify key molecular pathways, which, when compromised, might contribute to the onset of dystonia. Since primary dystonia seems to result from a chronic cellular dysfunction, rather than a degenerative process, there are greater grounds for the belief that it may eventually be possible to ameliorate the disease by correcting the underlying dysfunction in these pathways. With this in mind, what do the functions of the causative genes identified so far tell us about the pathophysiology of primary dystonia?

One inescapable observation, evident from even a cursory look at the genes mentioned in the preceding sections, is the apparent range of cellular pathways implicated in the pathogenesis of dystonia. This suggests that the phenotypic heterogeneity of the dystonias is matched by a similar heterogeneity of pathological mechanism and that dysfunction in distinct pathways or cellular components is capable of resulting in the common clinical manifestations of the disease. Nonetheless, several themes can be discerned on closer examination. Firstly, and perhaps unsurprisingly, dopamine signalling problems are clearly an important pathogenic mechanism in dystonia. All three of the genes involved in DOPA-responsive dystonia (*GCH1*, *TH* and *SPR*) are directly involved in the dopamine synthesis pathway (Fig. 2); *GNAL* encodes the alpha subunit of G protein that directly couples to D1 receptors; and knock-in mouse models of the GAG deletion in *TOR1A* have been shown to exhibit neurochemical and structural changes in the dopamine pathways of the brain (Song *et al.*, 2012). Regulation of gene expression and cell cycle dynamics also appear to be important: the protein products of *THAP1* and *TAF3* are both transcription factors, whereas that of *CIZ1* is a DNA replication factor that acts to transform nuclei poised to begin DNA synthesis into nuclei actively synthesizing DNA (Coverley *et al.*, 2005). With the recent identification of *TUBB4A* as the cause of whispering dysphonia, the function of several genes now point to cell structure as playing an important role in dystonia. *TUBB4A* encodes tubulin, the major component of the cytoskeleton; torsin A appears to be important for the structure of the nuclear envelope where it may form a bridge complex between it and the cytoskeleton (Atai *et al.*, 2012) and also interacts with vimentin, a type III intermediate filament important for motility, chemotaxis, adhesion, intracellular signalling and neurite outgrowth (Hewett *et al.*, 2006); and *SGCE*, a gene linked to myoclonus-dystonia, encodes a member of the dystrophin-glycoprotein complex that connects the cytoskeleton to the extracellular matrix in muscle and may play a role in synaptic organization in the CNS (Anastasi *et al.*, 2012). Synaptic dysfunction is also highlighted by the recent identification of *PRRT2*, the protein product of which interacts with the SNARE protein, SNAP25 (Lee *et al.*, 2012), in addition to evidence suggesting torsin A may regulate vesicular traffic and, by extension, neurotransmitter turnover (Warner *et al.*, 2010). Finally, the identification of mutations in *ANO3*, which encodes a Ca^2+^-gated chloride channel predominantly expressed in the striatum, as a cause of craniocervical dystonia suggests that altered neuronal excitability due to ion channel pathology could also result in dystonia and raises the possibility that medication aimed at correcting the kinetics of the channel might be an effective treatment in a subset of patients. *ATP1A3*, the cause of rapid onset dystonia-parkinsonism and alternating hemiplegia of childhood, encodes the catalytic subunit of a Na^+^/K^+^ pump that is expressed in neurons and cardiac cells and that is involved in maintaining ionic gradients (de Carvalho Aguiar *et al.*, 2004).

## The heredodegenerative dystonias

This category comprises a large number of complex neurological disorders of which dystonia can sometimes be a significant feature. However, it is important to recognize that dystonia may not be present in all cases or, more often still, may not be the dominant neurological sign within the clinical picture. Many of the conditions have a known genetic cause, but a full exposition of their clinical features and molecular basis is beyond the scope of this review. Instead, they are summarized in Table 7 by mode of inheritance.

**Table 7:** The major forms of heredodegenerative dystonia where a genetic cause has been identified, arranged by mode of inheritance

Disease	Gene	Major features besides dystonia
**Autosomal dominant**		
Dentatorubral-pallidoluysian atrophy	*ATN1*	Ataxia, chorea and cognitive decline.
Huntington’s disease	*IT-15*	Chorea, depression and cognitive decline.
Huntington’s disease-like 2	*JPH3*	Chorea, parkinsonism, ataxia and cognitive decline
Neuroferritinopathy	*FTL*	Chorea and parkinsonism.
SCA 2	*ATXN2*	Ataxia, ocular movement disorders, spasticity and parkinsonism
SCA 3	*ATXN3*	Ataxia, spasticity, parkinsonism and ocular movement disorders.
SCA 7	*ATXN7*	Ataxia, pigmentary macular degeneration, brisk reflexes
SCA 17	*TBP*	Chorea, parkinsonism, ataxia and cognitive decline
**Autosomal recessive**		
Ataxia-telangectasia	*ATM*	Ataxia, oculomotor apraxia, telangiectasia, susceptibility to malignancy.
Choreoacanthocytosis	*VPS13A*	Chorea, orofacial dyskinesias, cognitive decline, axonal neuropathy
Ceroid-lipofuscinosis	*Multiple genes*	Visual failure, cerebral atrophy and seizures
Dystonia with brain manganese deposition	*SLC30A10*	Hepatic cirrhosis, polycythemia and hypermanganesaemia.
Fucosidosis	*FUCA1*	Mental retardation, growth retardation, dysostosis multiplex, angiokeratoma
FBX07-associated neurodegeneration	*FBX07*	Parkinsonism
Glutaric acidaemia type 1	*GCDH*	Macrocepahaly, encephalopathic crises, axial hypotonia, parkinsonism.
Hereditary dopamine deficiency syndrome	*SLC6A3*	Pyramidal tract signs, eye movement disoreder, hyper and hypokinetic movement disorders
Kufor-Rakeb syndrome	*ATP13A2*	Parkinsonism
Infantile striatonigral degeneration	*NUP62*	Choreoathetosis, spasticity, nystagmus, developmental regression
Metachromatic leukodystrophy	*ARSA*	Mental retardation, spasticity and bulbar palsy
Mitochondrial membrane protein-associated neurodegeneration (MPAN)	*C19orf12*	Cognitive decline, prominent neuropsychiatric abnormalities, motor neuronopathy
Neimann Pick type C and D	*NPC1/NPC2*	Ataxia, ocular motor abnormalities (vertical gaze palsy), seizures
Neurodegeneration with brain iron accumulation type 1	*PANK2*	Parkinsonism, behavioural changes, pigmentary retinopathy in 50%
Neurodegeneration with brain iron accumulation type 2	*PLA2G6*	Parkinsonism, pyramidal signs, cognitive decline, cerebellar ataxia
Parkin-related parkinsonism	*PRKN*	Parkinsonism
Tay-Sach’s disease	*HEXA*	Psychomotor regression, dementia, blindness.
Wilson’s disease	*ATP7B*	Tremor, parkinsonism
**X-Linked**		
Dystonia-deafness syndrome	*TIMM8A*	Progressive hearing loss, spasticity, cortical blindness
Lesch-Nyhan syndrome	*HPRT*	Choreoathetosis, ballismus, cognitive and attentional deficits, self-injurious behaviours
Lubag disease (DYT3)	*TAF1*	Parkinsonism
Pelizaeus-Merzbacher disease	*PLP1*	Pyramidal dysfunction, cerebellar ataxia, head tremor
Rett syndrome	*MECP2*	Mental retardation, motor regression, autistic behaviours, seizures
Static encephalopathy of childhood with neurodegeneration in adulthood	*WDR45*	*De novo* inheritance pattern, global developmental delay, parkinsonism and dementia
**Mitochondrial**		
Leber’s optic neuropathy	*ND1, ND4, ND6*	Bilateral or sequential visual failure
Leigh syndrome	Multiple genes	Optic atrophy, ophthalmoplegia, ataxia, spasticity and developmental delay/regression. May also be autosomale recessive inheritance.
Myoclonic Epilepsy with Ragged Red Fibres (MERRF)	Mainly *tRNA(lys)* but others reported	Epilepsy, short stature, hearing loss

Major clinical features besides dystonia are indicated.

Notably, DYT3-related dystonia belongs to this category of disease and is perhaps worth a more detailed consideration. It is primarily found in Filipino males, due to a founder mutation, and is inherited in an X-linked recessive fashion. It is due to a 2.6 kb retrotransposon insertion in intron 32 of the *TAF1* (TATA box-binding protein-associated factor 1) gene (Makino *et al.*, 2007). The insertion appears to reduce neuron-specific expression of TAF1 and the dopamine receptor D2 gene in the caudate nucleus (Makino *et al.*, 2007; Muller, 2009). Symptoms start as focal dystonia and progress to multifocal/generalized dystonia, sometimes with parkinsonism. Neuronal degeneration on post-mortem analysis has been described in association with mutations in this gene (Waters *et al.*, 1993; Evidente *et al.*, 2002).

## The genetics of sporadic dystonia

To date, no genome-wide association study has been reported for sporadic dystonia. Previous experience in allied diseases, such as Parkinson’s disease or Alzheimer’s disease, suggests that to be successful such a study would need to be a large-scale, international effort that included thousands of case and control subjects. Such research is, of course, expensive and would require a considerable funding commitment. A second problem that a potential association study would face is defining its case cohort. Sporadic dystonia is an umbrella term, comprising multiple phenotypic subtypes that may well have distinct genetic architectures. In this respect, it is not unlike epilepsy, a condition for which large association studies have been somewhat disappointing (Kasperaviciute *et al.*, 2010). Difficult decisions will have to be made regarding whether to group all sporadic dystonia together and risk ‘diluting’ individual signals associated with particular subtypes or whether to spilt them and risk compromising the power of the studies to detect an association.

In the case of sporadic primary dystonia, only candidate gene association studies have been published. Six of these studies have examined common single nucleotide polymorphisms (SNPs) in and around the *TOR1A* gene. Two showed strong associations between individual SNPs and susceptibility for primary dystonia in populations from Iceland, southern Germany and Austria, and the United States (Clarimon *et al.*, 2005; Kamm *et al.*, 2006; Sharma *et al.*, 2010). However, others have shown only modest associations in Indian, Italian and northern German populations (Naiya *et al.*, 2006; Clarimon *et al.*, 2007; Bruggemann *et al.*, 2009) and yet other similar studies have been unable to replicate these associations (Sibbing *et al.*, 2003; Hague *et al.*, 2006). In contrast to *TOR1A*, no strong associations have been reported with polymorphisms in the *THAP1* gene (Xiao *et al.*, 2011; Kaffe *et al.*, 2012). One recent study employed a haplotype tagging strategy to cover the majority of common variability in *TOR1A*, *TAF1*, *GCH1*, *THAP1*, *MR-1*, *SGCE*, *ATP1A3* and *PRKRA* (Newman *et al.*, 2012). No association survived correction for multiple testing in this study, though three variants in *GCH1* did show significant association before correction and would merit follow-up in a larger case-control cohort.

## Conclusions

In recent years, advances in sequencing technology have accelerated the pace of gene discovery in neurology and the field of dystonia has been no exception, with several new genes appearing on the scene. Indeed, there is every reason to believe that the pace of discovery will continue to accelerate as sequencing costs fall and exome sequencing gives way to affordable whole-genome sequencing, allowing examination for the first time of non-coding and regulatory regions of DNA and their potential contributions to disease.

By pointing researchers in the direction of cellular pathways that are dysfunctional in familial dystonia, the discovery of new causative genes is the first step in unravelling the complex molecular pathophysiology that underlies not just familial but also, one would hope, sporadic forms of dystonia. Current data suggest that dystonia can result from dysfunction is a wide variety of cellular pathways, which probably reflects and in part explains the wide phenotypic variety seen in the disorder. Further functional work, particularly on those new genes that have only recently been published and still require independent confirmation, may help us identify a core set of pathogenic mechanisms in dystonia that might form the target for novel treatments. In this respect, induced pluripotent stem cell-derived neurons would offer the advantage of a more accurate model system, as well as an initial means of assessing the effect of any proposed treatments. Finally, it remains important to examine the genetics of sporadic dystonia independently by means of a large scale, genome-wide association study but, in order for this to be successful, international co-operation and a considerable funding commitment will be required.
